# Characterization of a Novel Ginsenoside MT1 Produced by an Enzymatic Transrhamnosylation of Protopanaxatriol-Type Ginsenosides Re

**DOI:** 10.3390/biom10040525

**Published:** 2020-03-31

**Authors:** Byeong-Min Jeon, Jong-In Baek, Min-Sung Kim, Sun-Chang Kim, Chang-hao Cui

**Affiliations:** 1 Department of Biological Sciences, Korea Advanced Institute of Science and Technology, 291 Daehak-Ro, Yuseong-Gu, Daejeon 305-701, Korea; jbm0901@kaist.ac.kr (B.-M.J.); baekji@kaist.ac.kr (J.-I.B.); mskim8906@kaist.ac.kr (M.-S.K.); 2 Intelligent Synthetic Biology Center, 291 Daehak-Ro, Yuseong-Gu, Daejeon 305-701, Korea; 3 KAIST Institute for Biocentury, Korea Advanced Institute of Science and Technology, 291 Daehak-Ro, Yuseong-Gu, Daejeon 305-701, Korea

**Keywords:** ginsenosides, dammarane-type triterpene saponin, ginsenoside MT1, transglycosylation, biotransformation, biotechnology

## Abstract

Background: Ginsenosides, triterpene saponins of *Panax* species, are considered the main active ingredients responsible for various pharmacological activities. Herein, a new protopanaxatriol-type ginsenoside called “ginsenoside MT1” is described; it was accidentally found among the enzymatic conversion products of ginsenoside Re. Method: We analyzed the conversion mechanism and found that recombinant β-glucosidase (MT619) transglycosylated the outer rhamnopyranoside of Re at the C-6 position to glucopyranoside at C-20. The production of MT1 by trans-rhamnosylation was optimized and pure MT1 was obtained through various chromatographic processes. Results: The structure of MT1 was elucidated based on spectral data: (20S)-3β,6α,12β,20-tetrahydroxydammarene-20-O-[*α*-L-rhamnopyranosyl(1→2)-β-D-glucopyranoside]. This dammarane-type triterpene saponin was confirmed as a novel compound. Conclusion: Based on the functions of ginsenosides with similar structures, we believe that this ginsenoside MT1 may have great potential in the development of nutraceutical, pharmaceutical or cosmeceutical products.

## 1. Introduction

Ginsenosides—triterpene saponins composed of a dammarane skeleton with several glycosylation positions—are generally considered the main active components of ginseng, that has been used as a traditional herbal medicine in East Asian countries for thousands of years to stimulate physical and mental activity [1,2]. Ginsenosides can be categorized as protopanaxadiol (PPD) and protopanaxatriol (PPT) saponins based on the structure of their aglycon. More than 100 kinds of ginsenosides have been found [3,4]; their pharmacological effects vary according to their attached sugars and aglycon structures [5,6,7]. Six major kinds of ginsenoside (Rb_1_, Rb_2_, Rc, Re, Rd and Rg_1_), as shown in Figure S1, constitute >90% of the total ginsenosides in ginseng [2,8]. They are relatively abundant in ginseng and can be converted into minor ginsenosides, which naturally exist in smaller amounts and have higher chemical reactivities [9,10,11].

Various methods have been reported for preparing minor ginsenosides with high conversion efficiency and few byproducts [12]. Many enzymes have been explored to efficiently convert major ginsenosides into pharmacologically active, rare minor ginsenosides [5,13,14]. Prior to this study, our research group cloned, characterized and applied many efficient glycoside hydrolases for the gram-scale production and purification of minor ginsenosides [15,16,17,18,19,20,21].

In our investigation into the enzymatic conversion of major ginsenosides, a new transformation product of ginsenoside Re that is different from the other hydrolyzing products (ginsenoside PPT, F_1_, Rg_1_ and Rg_2_) was accidentally found using a novel recombinant glucosidase (MT619) [15]. This compound was prepared at the gram scale using recombinant MT619 and isolated from the product mixture using chromatographic methods. Finally, the structure of this novel ginsenoside was elucidated based on spectral data.

## 2. Materials and Methods

### 2.1. Chemicals and Reagents

The standard forms of various ginsenosides (Rg_1_ and Re) used in the present study were purchased from Sigma Co., Ltd. (Louis, MO, USA). F_1_, Rg_2_(S), Rh_1_(S) and PPT were prepared as described in our previous study [22]. The other chemical reagents used were at least extra or better in quality than pure grade. Butanol, ethyl acetate, chloroform, ethanol and pyridine-d5 (Merck KGaA, Darmstadt, Germany) were purchased from Sam Chun Pure Chemical Co. (Pyeongtaek, Korea).

### 2.2. Biotransformation of Ginsenosides Using Recombinant MT619

Recombinant MT619 was expressed in *Escherichia coli* BL21 as described previously [15]. Briefly, the MT619-harboring pEX vector was inserted into the *E. coli* BL21 strain using heat-shock transformation. The cells grown in LB medium were supplemented with ampicillin at 37 °C until the culture reached an optical density of 600 nm (OD600) of 0.6, at which point protein expression was induced by adding 0.1 mM isopropyl-β-D-thiogalactopyranoside (IPTG). The bacterial cells were incubated further for 24 h at 18 °C and then harvested by centrifugation at 4000 rpm for 15 min. The cells were washed with 50 mM sodium phosphate and then resuspended in 50 mM sodium phosphate and 1% Triton X-100 (pH 7.0). The cells were disrupted by ultrasonication (Vibra-Cell; Sonics & Materials, Danbury, CT, USA). Intact cells and debris were removed by centrifugation at 13,000 rpm for 10 min to obtain a crude cell extract. His-tagged MT619 protein was purified by a HisTrap column (GE Healthcare, Menlo Park, California, USA). The 100 mM imidazole eluted protein was further purified by DEAE-cellulose DE-52 chromatography (Whatman, Maidstone, UK). Purified MT619 was used to examine its MT1 production efficiency by reacting with ginsenoside Re (2.0 mg/mL, pH 7.0) in a shaking incubator at 37 °C for 24 h. The ginsenosides in the samples were extracted with an equal volume of butanol and identified by thin layer chromatography (TLC).

### 2.3. Optimization of the Substrate Concentration

Ginsenoside MT1 production was evaluated using crude MT619 cell extracts. To determine the optimal concentration of selected substrates for the biotransformation reaction, cells were mixed with an equal volume of a substrate at 3.0–22.5 mg/mL at 37 °C. Samples were then withdrawn at regular intervals and analyzed by TLC.

### 2.4. Scaled-Up Ginsenoside MT1 Production

Production was scaled up to 1 L and a final concentration of 6.0 mg/mL substrate (ginsenoside Re). The transformation was performed by adding 300 mL of crude cell lysate in a shaking incubator at 200 rpm and 37 °C. After incubating for 24 h, the mixture was centrifuged at 4000 × *g* for 15 min and the supernatant was loaded into a column packed with HP20 resin (120 g) (Sigma-Aldrich, St Louis, MO, USA). One liter of water was used to remove unbound hydrophilic compounds and free sugar molecules, and the absorbed ginsenosides were eluted using three bed volumes of 95% ethanol. The eluted ethanol was evaporated in vacuo to produce dry powders.

### 2.5. Purification of MT1 from the Enzymatically Converted Products

Chromatographic pre-purification was performed with an LC-Forte/R system (YMC Korea Co. Ltd., Sungnam, Korea). One gram of the crude MT1 was dissolved in 20 mL of 10% methanol and centrifuged at 4000 × *g* for 20 min. After filtering with a syringe filter (0.2 µm), the dissolved sample was subjected to liquid chromatography (YMC-C18, 25 µm 120 g, 39 mm × 157 mm) and eluted with methanol-water (300 mL each at 5:5, 6:4, 7:3 and 8:2) to yield 13 fractions. The elution was fractionated every 100 mL, and the fractions containing MT1 were collected for purification.

### 2.6. Recycling Preparative High-Performance Liquid Chromatography (RPHPLC) Purification of MT1 from Biotransformed Products

The collected MT1 was further purified using RPHPLC (JAI NEXT Recycling Preparative HPLC LC-9210II NEXT, Japan Analytical Industry Co, Tokyo, Japan). RPHPLC was performed using a pre-packed column (JAIGEL-ODS-AP-L, 10 µm, 20 mm i.d. × 500 mm) purchased from Japan Analytical Industry Co. (Japan). The mobile phase was 50% acetonitrile; the flow rate was 7.0 mL/min. The MT1 solution was prepared by dissolving a sufficient quantity of the collected MT1 in 50% acetonitrile to give a final concentration of 35 mg/mL; 10 mL of the MT1 solution was loaded for purification.

### 2.7. High-Performance Liquid Chromatography (HPLC) Analysis

HPLC analysis of samples in the present study was performed using an Agilent 1260 Infinity HPLC system (Agilent Co., Santa Clara, CA, USA). Ginsenosides were separated on an YMC ODS C18 column (5 μm, 250 × 4.6 mm; YMC, Kyoto, Japan) with a guard column (Eclipse XDB C18, 5 μm, 12.5 × 4.6 mm; Agilent Co., Santa Clara, CA, USA). The gradient elution system consisted of water (A) and acetonitrile (B), and used the following gradient program: 0 → 10 min, 20% B; 10 → 40 min, 20 → 32% B; 40 → 48 min, 32 → 42% B; 48 → 60 min, 42 → 45% B; 60 → 83 min, 45 → 75% B; 83 → 85 min, 75 → 100% B; 85 → 95 min, 100% B; 95 → 95.01 min, 100 → 20% B; 95.01 → 100 min, 20% B. The detection wavelength was set to 203 nm and the flow rate was 1.6 mL/min.

### 2.8. Nuclear Magnetic Resonance (NMR) and Mass Spectrometry (MS) Analyses

NMR spectra were obtained using a Bruker AVANCE III 700 NMR spectrometer (Bruker, Rheinstetten, Germany) at 700 MHz (^1^H) and 175 MHz (^13^C), with chemical shifts given in ppm. Samples were separated with an Acquity UHPLC BEH C18 column (2.1 × 100 mm i.d., 1.7 μm) at room temperature. The mobile phases comprised deionized water containing 0.2% (v/v) formic acid (A) and acetonitrile (B). Quantitative analysis of the novel compound was performed using an LTQ-Orbitrap XL mass spectrometer (Thermo Scientific, Bremen, Germany) equipped with an ESI source (Thermo Electron, Bremen, Germany) in negative ion mode with a mass range of m/z 100–1500 Da. Optimal conditions were employed as follows: capillary voltage 20 V, capillary temperature 350 °C, spray voltage 3.5 kV and tube lens voltage 110 V.

### 2.9. Acidic Hydrolysis of MT1 and TLC Analysis

The purified compound (10 mg) was heated with 5% HCl mixture for 2 h at 70 °C. The residues from filtration and standard sugars were compared through cellulose TLC (Butanol:Ethanol:H_2_O, 50:50:30). TLC was conducted using 60F_254_ silica gel plates (Merck, Darmstadt, Germany) and CHCl_3_-CH_3_OH-H_2_O (65:35:10, v/v/v) as the developing solution. The results were visualized with 10% H_2_SO_4_ by heating at 110 °C for 5 min.

## 3. Results

### 3.1. Ginsenoside Re Transformation of MT619

**Figure 1:**
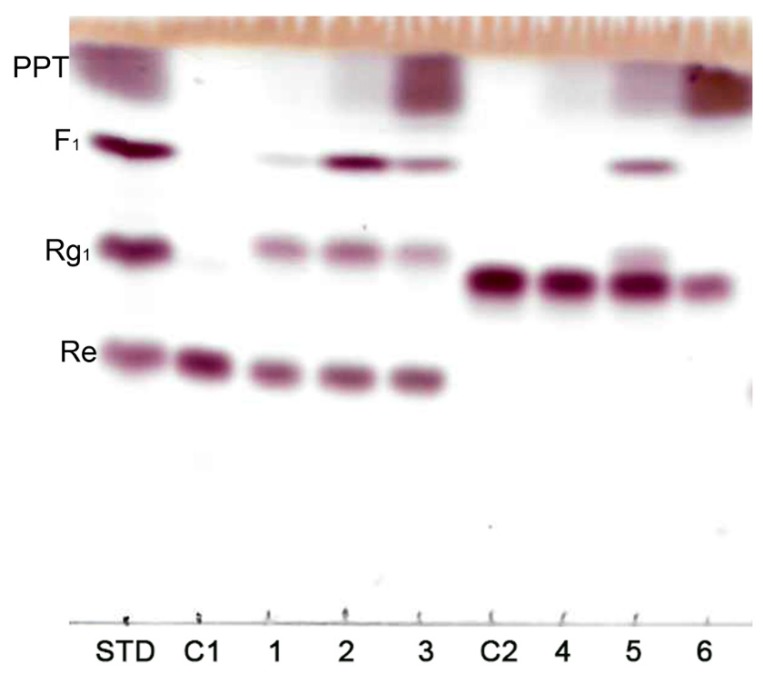
Thin layer chromatography of hydrolyzation products by MT619. C1, Re; 1, Re transformation products; 2, Re transformation products with F1 added; 3, Re transformation products with PPT added; C2, Rg_2_(S); 4, Rg_2_(S) transformation products; 5, Rg_2_(S) transformation products with F1 added; 6, Rg_2_(S) transformation products with PPT added.

MT619 was purified by Ni column and DEAE column chromatography as described in a previous study [15]. To analyze the transformation pathways, ginsenosides Re and Rg_2_ were reacted as substrates with purified MT619 ginsenoside and F_1_ and the conversion products were subjected to TLC analysis. The newly produced band (MT1), which had a different Rf value from those of ginsenoside Rg_1_ and Rg_2_, was identified from Re reaction mixtures (Figure 1). Rg_2_(S) was also hydrolyzed by MT619 into PPT, but MT1 was not reported. Interestingly, MT1 was only found in the Rg_2_ mixture containing F_1_ (Sample 5 in Figure 1), which clearly suggests that F_1_ is the recipient in trans-rhamnosyl reactions. No MT1 was formed in the PPT conversion mixture, suggesting that PPT cannot receive rhamnose. Rg_1_ and Rh_1_ were not found in the Re and Rg_2_ conversion mixtures; the existence of rhamnose was inferred by the rapid decomposition of Rg_1_ and Rh_1_ by MT619 into F_1_ and PPT. Released glucose and rhamnose moieties were also identified in the Re and Rg_2_ mixtures (Figure S2).

### 3.2. Optimization for Ginsenoside MT1 Production

**Figure 2:**
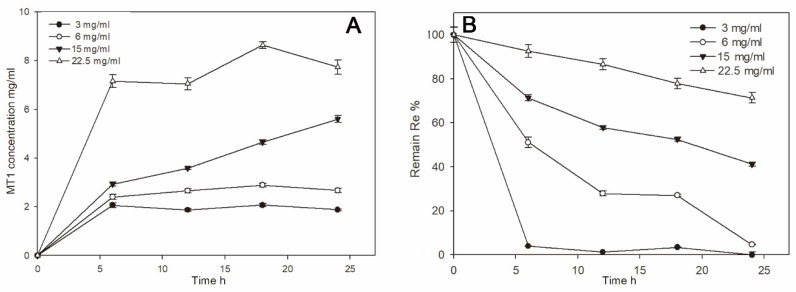
Effects of the substrate Re concentrations (**A**) and remaining Re concentrations (**B**) in the reaction mixture.

Optimal conditions for the biotransformation of Re into MT1 were determined by examining the substrate concentrations in the reaction mixture. Enzyme reactions were performed using of ginsenoside Re, which is one of the major ginsenosides in ginseng [23,24]. The concentration of MT1 increased in proportion to the substrate concentration (Figure 2A); the maximum concentration of MT1 produced was 8.0 mg/mL with 22.5 mg/mL Re 24 h after reaction. Note that >41.2% of Re remained after 24 h when the Re concentration was >15.0 mg/mL (Figure 2B); however, nearly all the Re was converted when the Re concentration was 6.0 mg/mL. Therefore 6.0 mg/mL Re was used for the scaled-up production of MT1.

### 3.3. Mass Production of MT1

**Figure 3:**
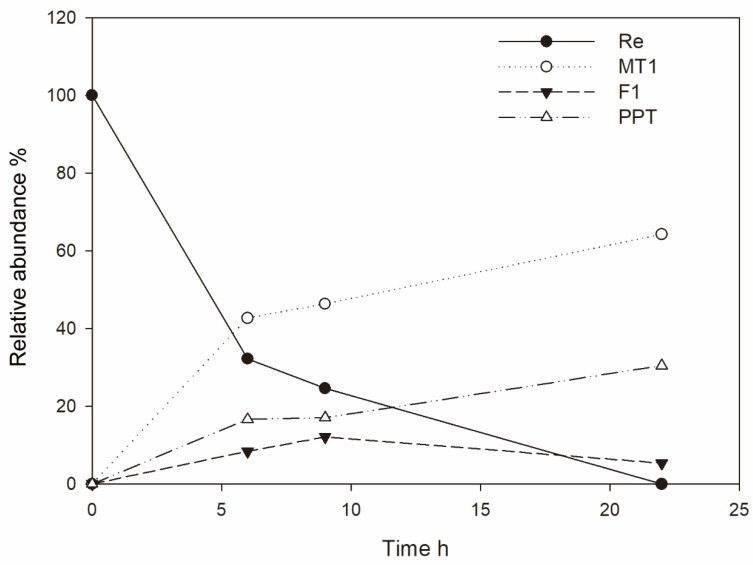
The relative abundance of ginsenosides Re, MT1, F1 and PPT in a scaled-up production reactor over time.

**Figure 4:**
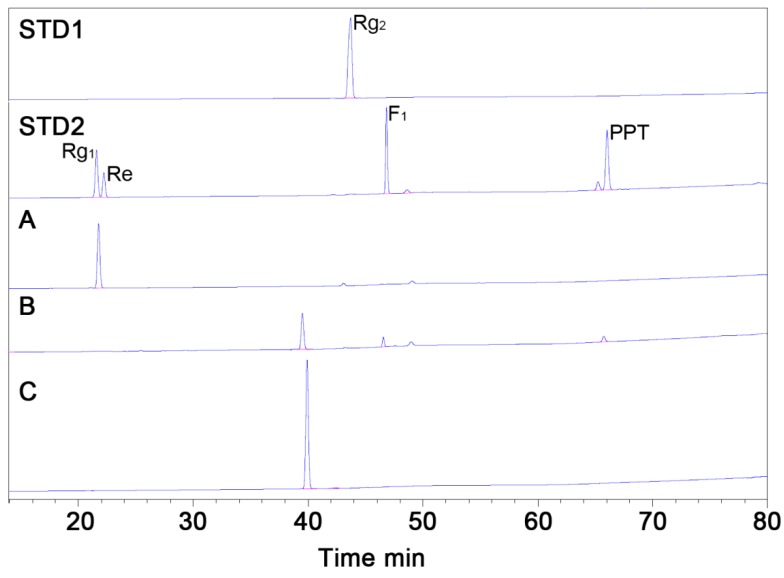
HPLC analysis of purified MT1 from the product mixture. (STD1), ginsenoside Rg_2_(S) standard; (STD2), standards of ginsenosides Rg_1_, Re, F_1_ and PPT; (**A**), substrate ginsenoside Re before purification; (**B**), biotransformed products from the MT619 conversion mixture; (**C**), MT1 isolated using Recycling preparative high-performance liquid chromatography (RPHPLC).

Six grams of Re in 1.0 L of phosphate buffer (pH 7.0) was reacted with cell lysates containing MT619. Re was completely converted 24 h after adding MT619 (Figure 3; Figure 4B). To remove proteins and impurities, the supernatants of the reaction mixture were applied to a 120 g HP20 macroporous resin. After washing with water, ethanol was used to elute the ginsenosides from the resin. The eluate was then evaporated in vacuo, yielding 3.8 g of mixed dry ginsenosides. In preparation for further purification, 1.0 g MT1 was dissolved in 200 mL of 10% methanol; the undissolved precipitants were separated using an ODS column to yield 13 fractions (Figure S3). MT1 was eluted in the 70% methanol fractions (9 and 10), yielding 655 mg of dry compounds.

### 3.4. Purification of MT1 using RPHPLC

Recycling preparative HPLC, which can enhance the separation of compounds by recycling the effluent sample many times over the column without increasing the length of the chromatographic column, was used for further purification. MT1 was purified from the product mixture with a preparative ODS column by RPHPLC. Dry MT1 mixture (300 mg) was loaded onto the RPHPLC column for separation. MT1 was baseline-resolved after three effective columns (Figure S4). The purification process resulted in 214 mg of MT1 with 98.9% chromatographic purity (Figure 4C). The total yield of the production of MT1 from Re was 47.4%.

### 3.5. Structural Characterization of Ginsenoside MT1

Pure ginsenoside MT1 was obtained as a white powder with a molecular ion peak at m/z 785.5 in the positive LC-MS, which corresponds to the molecular formula C_42_H_72_O_13_ (Figure S5). Acidic hydrolysis of the compound yielded neohesperidose, the signals of which were similar to those of other neohesperidose-containing compounds, such as ginsenoside Rg_2_(S) and neohesperidin (Figure S6).

Structural characterization was performed by a Bruker AVANCE III 700 NMR spectrometer operating at 700 MHz (^1^H) and 175 MHz (^13^C) with chemical shifts given in ppm for atomic force microscopy (AFM) to determine the chemical structure of MT1 (Table 1). The ^1^H-NMR spectrum showed one olefinic (δH 5.28), two anomeric (δ 5.25 and 6.61) protons and a methyl proton signal (δ 1.83) like in L-rhamnopyranoside. Coupling constants suggested the configuration of the anomeric positions to be α-and-β form for the anomeric proton signals in the ^1^H-NMR spectrum. The ^13^C-NMR signals of the sugar were observed as one hemiacetal (δ_C_ 96.7), four oxygenated methines (δ_C_ 79.8, 78.4, 76.6 and 71.6) and one oxygenated methylene (δ_C_ 62.95), indicating that the sugar is b-glucopyranose.

**Figure 5:**
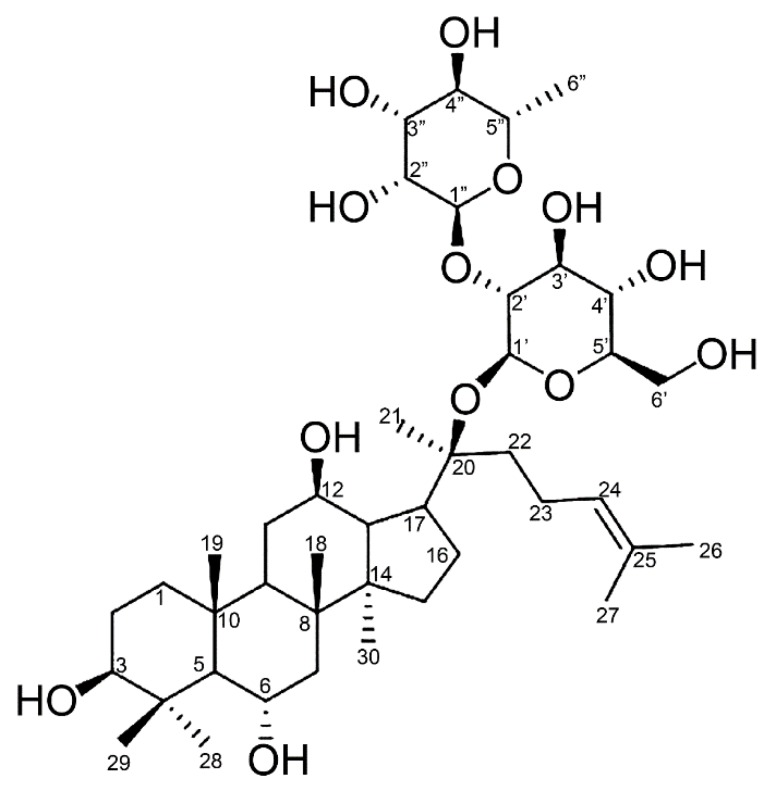
The structure ginsenoside MT1 based on the spectral data.

The β-D-glucopyranosyl anomeric proton signals were confirmed to be linked at the C-20 position by long-range heteronuclear multiple bond connectivity (HMBC) correlations between the proton signal at δ 5.25 (H-1”) and the carbon signal at δ 84.0 (C-20). A large downfield shift in the C-2” (δ 76.6) was observed for the inner β-D-glucopyranosyl moiety at C-20 of the aglycone, which showed that the C-1’’’ (δ 6.61) in the terminal α-L-rhamnopyranosyl moiety is linked to the inner β-D-glucopyranosyl moiety at C-20 [25]. In summary, these results indicate that the metabolite of MT619 is (20S)-3β,6α,12β,20-tetrahydroxydammarene-20-O-[α-L-rhamnopyranosyl(1→2)-β-D-glucopyranoside] (Figure 5) or ginsenoside MT1.

**Figure 6:**
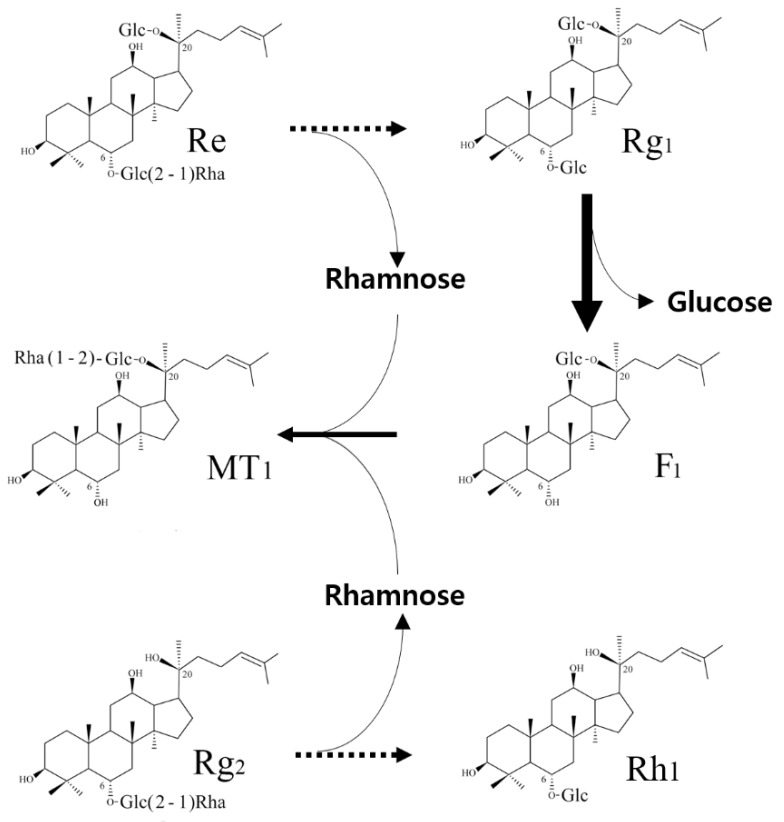
Conversion pathways of ginsenosides Re and Rg_2_(S) by MT619.

Based on the above experimental results, the conversion pathways were deduced as shown in Figure 6. In the conversion of Re, the rhamnopyranoside moieties were released from the substrates and transformed into the C-20 outer glucopyranoside of ginsenoside F_1_. When MT619 reacted with Rg_2_, the rhamnosyl transformation could only occur with the existence of the attached glucopyranoside at C-20 positions such as F_1_. The mechanisms of rhamnose transformation by MT619 warrant further exploration.

## 4. Discussion

Many glycoside hydrolases have been shown to possess transglycosylation capabilities in addition to hydrolytic activity [26]. In substrate transglycosylation of glycoside hydrolases, the glycosyl part of the substrate is transferred to a hydroxyl-containing compound instead of water moieties [27]. Some GH family 3 enzymes also show transglycosylation capabilities from various substrates. A GH3 cellobiase from *Phanerochaete chrysosporium* can hydrolyze and transglycosylate glucopyranoside from laminarioligosaccharides [28]. β-glucosidase from *Aspergiluus niger* strain ASKU28 also showed transglucosidase activity against various substrates [29]. MT619 has been classified into GH family 3 according to the Carbohydrate-Active enZymes (CAZy). To the best of our knowledge, this is the only transrhamnosylation activity reported in glycoside hydrolase family 3 to date. Further studies are needed to reveal its sequence-activity relevance.

Some glycosylated ginsenoside derivatives from various natural ginsenosides have been synthesized using enzymatic methods. A novel α-glycosylated ginsenoside F_1_((20S)-3β,6α,12β-trihydroxydammar-24ene-(20-O-β-D-glucopyranosyl-(1→2)-α-D-glucopyranoside)) was synthesized using a cyclodextrin glucanotransferase [30]; ginsenoside Ia((20S)-3b,6a,12b,20-tetrahydroxydammar-24-ene-20-O-b-D-glucopyranosyl-3-O-b-D-glucopyranoside) was synthesized using UDP-glycosyltransferase (BSGT1) from ginsenoside F_1_ [31]. In addition, various Rg_1_ a-glycosylated ginsenosides (α-1,3-, α-1,4- or α-1,6-glucosidic linkage in β-glucose moieties) linked at C-6 and C-20 positions of aglycones have been synthesized using rice seed a-glucosidases [32], although no trans-rhamnosyl activity has been found using ginsenosides as rhamnose receptors.

A similar ginsenoside—ginsenoside Rg_18_ (Figure S1), which differs from MT1 in that it is linked to an additional glucose moiety at the C-6 position—was recently found, isolated and characterized from *Panax ginseng* roots [33]. Rg_18_ has been shown to have various important pharmacological effects, such as anticancer [34] and neuroprotective effects [35]. However, the content of Rg_18_ in ginseng is <36.0 µg/g of ginseng, which means that its application is limited by its scarcity in plants. Fortunately, MT1 can be produced efficiently from the relatively abundant ginsenoside Re using the recombinant MT619 with high recovery, which can make it industrially applicable. Furthermore, the deglycosylated ginsenosides generally have shown enhanced pharmacological effects and absorbances than the original ginsenosides [36,37,38]. Our group is drawing on the properties of MT1 to conduct experiments on compounds with higher anticancer, anti-inflammatory, anti-oxidation and anti-aging.

## 5. Conclusions

In summary, this paper describes characterization and mass production of a new dammarane-type triterpene saponin from enzymatic converted products. The trans-rhamnosyl activity of MT619 against ginsenoside Re to F_1_ resulted in the new ginsenoside MT1, a PPT-type ginsenoside containing one neohesperidose moiety at the C-20 position (confirmed based on spectral data). This unknown compound can be efficiently produced from abundant ginsenoside Re using MT619. Various studies are on-going by our group to investigate its pharmacological effects. We expect potential commercial uses of MT1 in nutraceutical, pharmaceutical or cosmeceutical areas.

## Figures and Tables

**Table 1:** ^1^H- (700 MHz) and ^13^C-NMR (175 MHz) spectra of MT1.

Aglycon Moiety Position	δ_H_ mult., (*J* Hz)	δ_C_ mult.	Sugar Moiety Position	δ_H_ mult., (*J* Hz)	δ_C_ mult.
1	1.73 m, 1.06 m	39.4 t	Glc-1′	5.25 d (6.3)	96.7 d
2	1.93 m, 1.88 m	28.2 t	Glc-2′	4.29*^b^*	76.6 d
3	3.56 t-like (5.6)	78.4 d	Glc-3′	4.29*^b^*	79.8 d
4		40.4 s	Glc-4′	4.09 t (9.1)	71.6 d
5	1.27 d (10.5)	61.8 d	Glc-5′	3.88 m	78.4 d
6	4.40*^c^*	67.8 d	Glc-6′	4.40*^c^*, 4.25 m	62.5 t
7	2.04 m, 1.90 m	47.5 t	Rha-1″	6.61 s	101.4 d
8		41.2 s	Rha-2″	4.79 brs	72.5 d
9	1.67 m	49.7 d	Rha-3″	4.64 t (4.9)	72.6 d
10		39.3 s	Rha-4″	4.40*^c^*	74.2 d
11	2.20 m, 1.55 m	31.1 t	Rha-5″	4.89 dd (9.1, 5.6)	69.4 d
12	4.11 m	70.7 d	Rha-6″	1.83 d (5.6)	19.0 q
13	2.00 m	49.0 d	3′-OH	7.50 brs	
14		51.6 s	4′-OH	7.40 d (4.2)	
15	1.58 m, 1.03 m	30.9 t	6′-OH	5.90 brs	
16	1.95 m, 1.44 m	26.7 t	2″-OH	6.76 d (4.2)	
17	2.77 dd (18.2, 10.5)	53.3 d	3″-OH	6.46 brs	
18	1.12 s	17.4 q	4″-OH	6.81 d (3.5)	
19	1.05 s	17.5 q			
20		84.0 s			
21	1.59 s	22.8 q			
22	2.45 m, 1.98 m	35.9 t			
23	2.29 m	24.0 t			
24	5.28*^d^*	126.0 d			
25		130.8 s			
26	1.62 s	25.8 q			
27	1.65 s	17.9 q			
28	2.02 s	32.0 q			
29	1.48 s	16.5 q			
30	1.17 s	17.2 q			
3-OH	5.76 d (5.6)				
6-OH	5.28*^d^*				
12-OH	5.58 s				

*^a^* Measured at 700 and 175 MHz; obtained in C5D5N with TMS as an internal standard. The assignments were based on ^1^H-^1^HCOSY, HSQC, and HMBC experiments. ^*b*–*d*^ Overlapped with other signals.

## Supplementary Materials

The following are available online at https://www.mdpi.com/2218-273X/10/4/525/s1, Figure S1: Chemical structures of ginsenosides; Figure S2. Released glucose (orange arrow) and rhamnose (blue arrow)s in the supernatant of the reaction products of Re and Rg_2_(S); Figure S3. Thin layer analysis of the fractions for the purification of MT1 using the ODS column; Figure S4. RPHPLC chromatogram showing the resolution of the Rb3 and Rd; Figure S5. Positive-ion mode HPLC-ESI-MS/MS of the molecular ion (sodium adduct) of MT1; Figure S6. TLC analysis of acidic hydrolysis products from neohesperidose-moiety harboring substrates by hydrogen chloride.

## References

[1] Chung H.S., Lee Y.C., Rhee Y.K., Lee S.Y. (2011). Consumer acceptance of ginseng food products. J. Food Sci..

[2] Shi Y., Sun C.J., Zheng B., Gao B., Sun A.M. (2013). Simultaneous Determination of Ten Ginsenosides in American Ginseng Functional Foods and Ginseng Raw Plant Materials by Liquid Chromatography Tandem Mass Spectrometry. Food Anal. Methods.

[3] Jia L., Zhao Y.Q. (2009). Current Evaluation of the Millennium Phytomedicine-Ginseng (I): Etymology, Pharmacognosy, Phytochemistry, Market and Regulations. Curr. Med. Chem..

[4] Leung K.W., Leung F.P., Mak N.K., Tombran-Tink J., Huang Y., Wong R.N. (2009). Protopanaxadiol and protopanaxatriol bind to glucocorticoid and oestrogen receptors in endothelial cells. Brit. J. Pharmacol..

[5] Wong A.S.T., Che C.M., Leung K.W. (2015). Recent advances in ginseng as cancer therapeutics: A functional and mechanistic overview. Nat. Prod. Rep..

[6] Biswas T., Mathur A.K., Mathur A. (2017). A literature update elucidating production of Panax ginsenosides with a special focus on strategies enriching the anti-neoplastic minor ginsenosides in ginseng preparations. Appl. Microbiol. Biot..

[7] Hao H.P., Zheng X., Wang G.J. (2014). Insights into drug discovery from natural medicines using reverse pharmacokinetics. Trends Pharmacol. Sci..

[8] Zhou S.S., Xu J.D., Zhu H., Shen H., Xu J., Mao Q., Li S.L., Yan R. (2014). Simultaneous determination of original, degraded ginsenosides and aglycones by ultra high performance liquid chromatography coupled with quadrupole time-of-flight mass spectrometry for quantitative evaluation of Du-Shen-Tang, the decoction of ginseng. Molecules.

[9] Kim W.Y., Kim J.M., Han S.B., Lee S.K., Kim N.D., Park M.K., Kim C.K., Park J.H. (2000). Steaming of ginseng at high temperature enhances biological activity. J. Nat. Prod..

[10] Bae E.A., Han M.J., Kim E.J., Kim D.H. (2004). Transformation of ginseng saponins to ginsenoside Rh2 by acids and human intestinal bacteria and biological activities of their transformants. Arch. Pharm. Res..

[11] Yun T.K., Lee Y.S., Lee Y.H., Kim S.I., Yun H.Y. (2001). Anticarcinogenic effect of Panax ginseng CA Meyer and identification of active compounds. J. Korean Med. Sci..

[12] Cui C.H., Kim S.C., Im W.T. (2013). Characterization of the ginsenoside-transforming recombinant beta-glucosidase from Actinosynnema mirum and bioconversion of major ginsenosides into minor ginsenosides. Appl. Microbiol. Biot..

[13] Shin K.-C., Oh D.-K. (2016). Classification of glycosidases that hydrolyze the specific positions and types of sugar moieties in ginsenosides. Crit. Rev. Biotechnol..

[14] Park C.S., Yoo M.H., Noh K.H., Oh D.K. (2010). Biotransformation of ginsenosides by hydrolyzing the sugar moieties of ginsenosides using microbial glycosidases. Appl. Microbiol. Biotechnol..

[15] Cui C.-h., Jeon B.-M., Fu Y., Im W.-T., Kim S.-C. (2019). High-density immobilization of a ginsenoside-transforming β-glucosidase for enhanced food-grade production of minor ginsenosides. Appl. Microbiol. Biotechnol..

[16] An D.-S., Cui C.-H., Siddiqi M.Z., Yu H.S., Jin F.-X., Kim S.-G., Im W.-T. (2017). Gram-Scale Production of Ginsenoside F1 Using a Recombinant Bacterial β-Glucosidase. Appl. Microbiol. Biotechnol..

[17] Cui C.-H., Fu Y., Jeon B.-M., Kim S.-C., Im W.-T. (2019). Novel enzymatic elimination method for the chromatographic purification of ginsenoside Rb3 in an isomeric mixture. J. ginseng Res..

[18] Siddiqi M.Z., Cui C.H., Park S.K., Han N.S., Kim S.C., Im W.T. (2017). Comparative analysis of the expression level of recombinant ginsenoside-transforming beta-glucosidase in GRAS hosts and mass production of the ginsenoside Rh2-Mix. PLoS ONE.

[19] Cui C.H., Kim J.K., Kim S.C., Im W.T. (2014). Characterization of a ginsenoside-transforming beta-glucosidase from Paenibacillus mucilaginosus and its application for enhanced production of minor ginsenoside F(2). PLoS ONE.

[20] Park M.K., Cui C.H., Park S.C., Park S.K., Kim J.K., Jung M.S., Jung S.C., Kim S.C., Im W.T. (2014). Characterization of recombinant beta-glucosidase from Arthrobacter chlorophenolicus and biotransformation of ginsenosides Rb1, Rb 2, Rc, and Rd. J. Microbiol..

[21] Du J., Cui C.H., Park S.C., Kim J.K., Yu H.S., Jin F.X., Sun C., Kim S.C., Im W.T. (2014). Identification and characterization of a ginsenoside-transforming beta-glucosidase from Pseudonocardia sp. Gsoil 1536 and its application for enhanced production of minor ginsenoside Rg2(S). PLoS ONE.

[22] Cui C.H., Liu Q.M., Kim J.K., Sung B.H., Kim S.G., Kim S.C., Im W.T. (2013). Identification and characterization of a Mucilaginibacter sp. strain QM49 beta-glucosidase and its use in the production of the pharmaceutically active minor ginsenosides (S)-Rh1 and (S)-Rg2. Appl. Environ. Microbiol..

[23] Ren G., Chen F. (1999). Simultaneous quantification of ginsenosides in American ginseng (Panax quinquefolium) root powder by visible/near-infrared reflectance spectroscopy. J. Agric. Food Chem..

[24] Wang Y., Pan J.Y., Xiao X.Y., Lin R.C., Cheng Y.Y. (2006). Simultaneous determination of ginsenosides in Panax ginseng with different growth ages using high-performance liquid chromatography-mass spectrometry. Phytochem. Anal..

[25] Iwamoto M., Fujioka T., Okabe H., Mihashi K., Yamauchi T. (1987). Studies on the Constituents of Actinostemma-Lobatum Maxim.1. Structures of Actinostemmoside-a, Actinostemmoside-B, Actinostemmoside-C, Actinostemmoside-D, Dammarane Triterpene Glycosides Isolated from the Herb. Chem. Pharm. Bull..

[26] Bissaro B., Monsan P., Faure R., O’Donohue M.J. (2015). Glycosynthesis in a waterworld: New insight into the molecular basis of transglycosylation in retaining glycoside hydrolases. Biochem. J..

[27] Geronimo I., Payne C.M., Sandgren M. (2018). Hydrolysis and Transglycosylation Transition States of Glycoside Hydrolase Family 3 beta-Glucosidases Differ in Charge and Puckering Conformation. J. Phys. Chem. B.

[28] Kawai R., Igarashi K., Kitaoka M., Ishii T., Samejima M. (2004). Kinetics of substrate transglycosylation by glycoside hydrolase family 3 glucan (1 -> 3)-beta-glucosidase from the white-rot fungus Planerochaete chrysosporium. Carbohyd. Res..

[29] Thongpoo P., McKee L.S., Araujo A.C., Kongsaeree P.T., Brumer H. (2013). Identification of the acid/base catalyst of a glycoside hydrolase family 3 (GH3) beta-glucosidase from Aspergillus niger ASKU28. Bba-Gen. Subjects.

[30] Moon S.S., Lee H.J., Mathiyalagan R., Kim Y.J., Yang D.U., Lee D.Y., Min J.W., Jimenez Z., Yang D.C. (2018). Synthesis of a Novel alpha-Glucosyl Ginsenoside F1 by Cyclodextrin Glucanotransferase and Its In Vitro Cosmetic Applications. Biomolecules.

[31] Wang D.D., Jin Y., Wang C., Kim Y.J., Perez Z.E.J., Baek N.I., Mathiyalagan R., Markus J., Yang D.C. (2018). Rare ginsenoside Ia synthesized from F1 by cloning and overexpression of the UDP-glycosyltransferase gene from Bacillus subtilis: Synthesis, characterization, and in vitro melanogenesis inhibition activity in BL6B16 cells. J. Ginseng. Res..

[32] Kim M.J., Kim Y.H., Song G.S., Suzuki Y., Kim M.K. (2016). Enzymatic transglycosylation of ginsenoside Rg1 by rice seed alpha-glucosidase. Biosci. Biotech. Bioch..

[33] Lee D.G., Quilantang N.G., Lee J.S., Geraldino P.J.L., Kim H.Y., Lee S.H. (2018). Quantitative Analysis of Dammarane-type Ginsenosides in Different Ginseng Products. Nat. Prod. Sci..

[34] Leem D.G., Shin J.S., Kim K.T., Choi S.Y., Lee M.H., Lee K.T. (2018). Dammarane-type triterpene ginsenoside-Rg18 inhibits human non-small cell lung cancer A549 cell proliferation via G1 phase arrest. Oncol. Lett..

[35] Kim M., Choi S.Y., Kim K.T., Rhee Y.K., Hur J. (2017). Ginsenoside Rg18 suppresses lipopolysaccharide-induced neuroinflammation in BV2 microglia and amyloid-beta-induced oxidative stress in SH-SY5Y neurons via nuclear factor erythroid 2-related factor 2/heme oxygenase-1 induction. J. Funct. Foods.

[36] Wang C.Z., Zhang B., Song W.X., Wang A., Ni M., Luo X., Aung H.H., Xie J.T., Tong R., He T.C. (2006). Steamed American ginseng berry: Ginsenoside analyses and anticancer activities. J. Agric. Food Chem..

[37] Wang J.R., Yau L.F., Zhang R., Xia Y., Ma J., Ho H.M., Hu P., Hu M., Liu L., Jiang Z.H. (2014). Transformation of ginsenosides from notoginseng by artificial gastric juice can increase cytotoxicity toward cancer cells. J. Agric. Food Chem..

[38] Qu Y., Liu H.-Y., Guo X.-X., Luo Y., Wang C.-X., He J.-H., Xu T.-R., Yang Y., Cui X.-M. (2018). Converting ginsenosides from stems and leaves of Panax notoginseng by microwave processing and improving their anticoagulant and anticancer activities. RSC Adv..

