# Supplementary material for: Characterization of a Novel Ginsenoside MT1 Produced by an Enzymatic Transrhamnosylation of Protopanaxatriol-Type Ginsenosides Re
Source: Biomolecules. 2020 Mar 31;10(4):525. doi: 10.3390/biom10040525 (PMC7226242; doi:10.3390/biom10040525)
Supplement: Supplementary file 1 [file biomolecules-10-00525-s001.pdf]

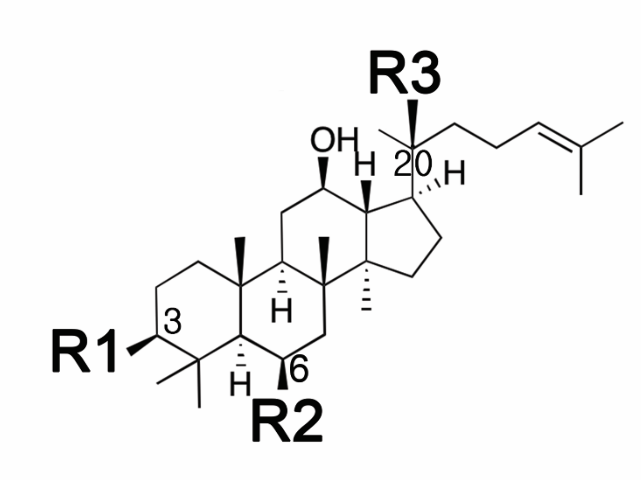

Ginsenoside	R1	R2	R3
Rb_1	Glc(1→2)Glc-	H-	Glc(1→6)Glc-
Rb_2	Glc(1→2)Glc-	H-	Arap(1→6)Glc-
Rd	Glc(1→2)Glc-	H-	Glc-
Rc	Glc(1→2)Glc-	H-	Araf(1→6)Glc-
Re	HO-	Rha(1→2)-Glc-	Glc-
Rg_2	HO-	Rha(1→2)-Glc-	HO-
Rg_1	HO-	Glc-	Glc-
Rh_1	HO-	Glc-	HO-
F_1	HO-	HO-	Glc-
PPT	HO-	HO-	HO-
Rg_{18}	HO-	Glc-	Rha(1→2)-Glc-
MT1	HO-	HO-	Rha(1→2)-Glc-

Glc: glucopyranoside; Arap: arabinopyranoside; Araf: arabinofuranoside; Rha: rhamnopyranoside

Figure. S1. Chemical structures of ginsenosides.

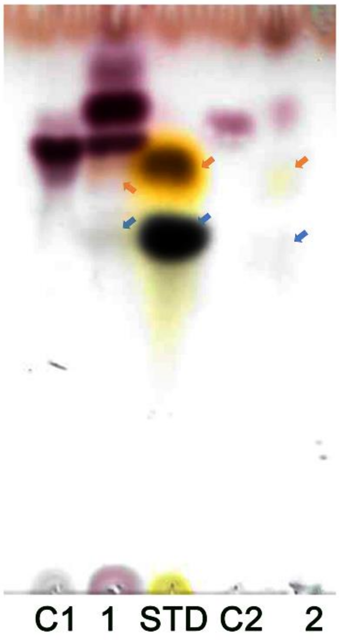

Figure S2. Released glucose(orange arrow) and rhamnose(blue arrow)s in the supernatant of

the reaction products of Re and Rg_2(S). C1, Re; 1, the MT619-hydrolyzed products of ginsenoside Re; C2, Rg_2(S); 2, the MT619-hydrolyzed products of ginsenoside Rg_2(S).

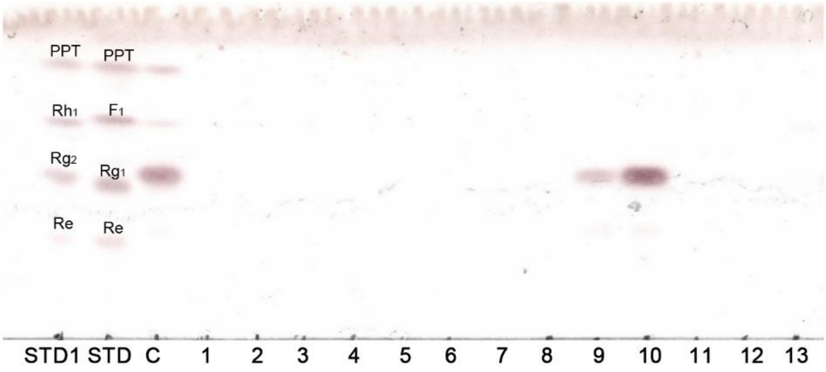

Figure S3. Thin layer analysis of the fractions for the purification of MT1 using the ODS column. The dissolved ginsenoside MT1 sample was subjected to the ODS column and eluted with the methanol-water solution to yield 13 fractions. The elution was fractionated every 100 mL, and 2µl of each fraction was loaded on the plate.

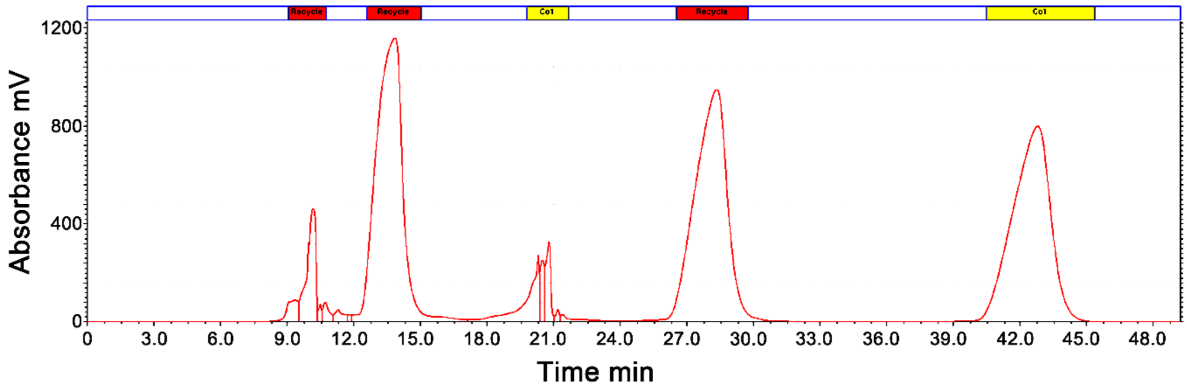

Figure S4. RPHPLC chromatogram showing the resolution of the Rb_3 and Rd. Note that these two peaks can be separated without recycling. Conditions: column, JAIGEL-ODS-AP-L (20 mm (i.d.) × 500 mm (l)); mobile phase: H_2O (solvent A) and acetonitrile (solvent B), isocratic elution: 40% B; flow rate, 7 mL/min, detection wavelength, 203 nm; sample loading concentration of 35 mg/mL; loading volume of 10 mL.

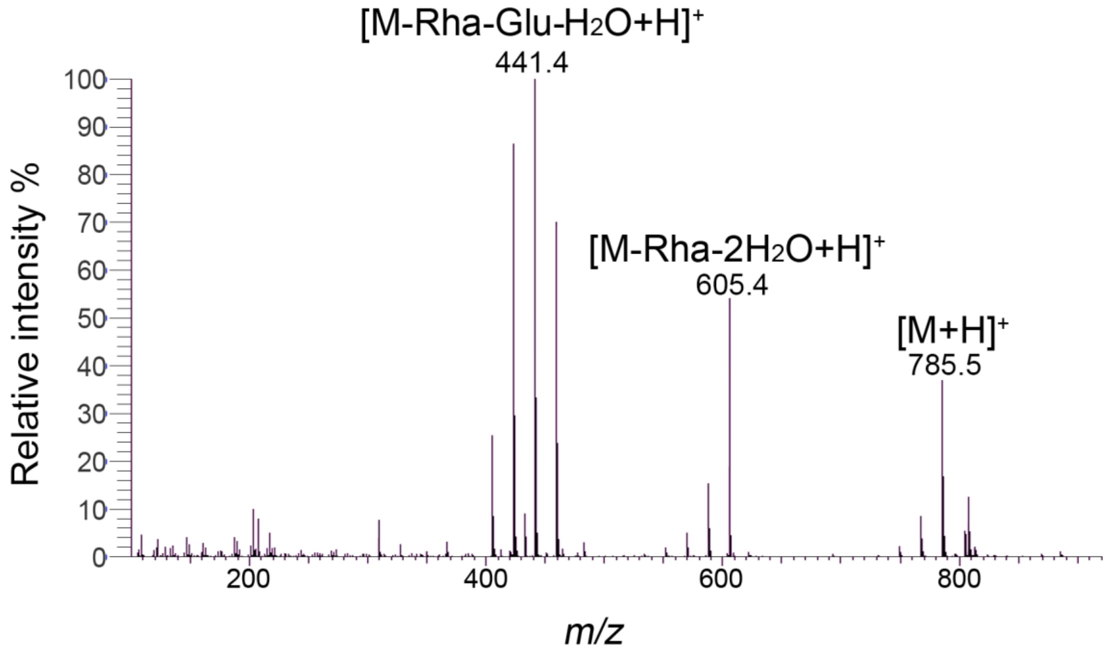

Figure S5. Positive-ion mode HPLC-ESI-MS/MS of the molecular ion (sodium adduct) of MT1. The detected fragments and their m/z values are shown at the top of the peaks.

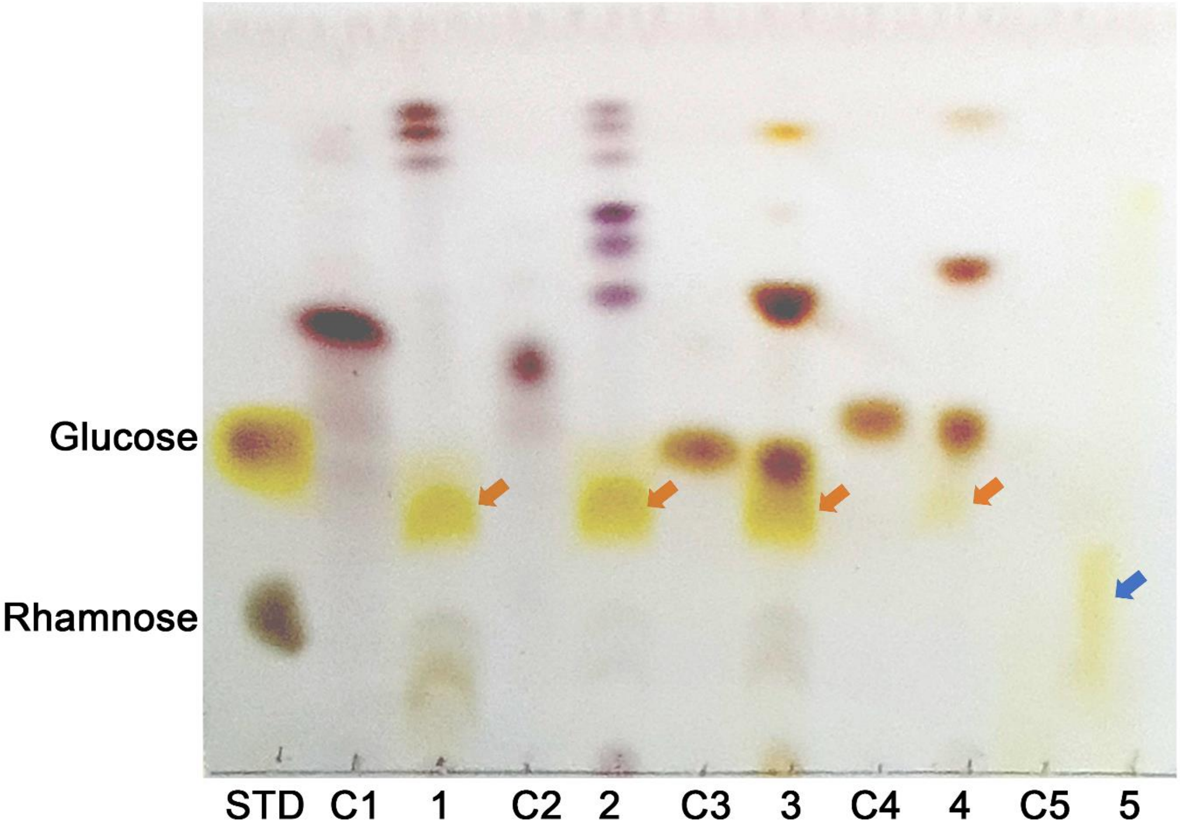

Figure S6. TLC analysis of acidic hydrolysis products from neohesperidose-moiety harboring substrates by hydrogen chloride. STD, standards of glucose and rhamnose moieties; C1, MT1; 1, acidic hydrolyzed products of MT1; C2, Rg_2(S); 2, acidic hydrolyzed products of Rg_2(S); C3, naringin; 3, acidic hydrolyzed products of naringin; C4, neohesperidin; 4, acidic hydrolyzed products of neohesperidin; C5, rutin; 5, acidic hydrolyzed products of rutin. The released neohesperidose (2-O-α-L-Rhamnopyranosyl-β-D-Glucopyranose)(orange arrow) from ginsenoside MT1, ginsenoside Rg_2(S), naringin, neohesperidose showed different Rf values with the released and rutinose (6-O-α-L-Rhamnopyranosyl-β-D-Glucopyranose)(blue arrow) from rutin.
